# Development and validation of prediction models for sentinel lymph node status indicating postmastectomy radiotherapy in breast cancer: population-based study

**DOI:** 10.1093/bjsopen/zraf047

**Published:** 2025-04-08

**Authors:** Miriam Svensson, Pär-Ola Bendahl, Sara Alkner, Emma Hansson, Lisa Rydén, Looket Dihge

**Affiliations:** Department of Clinical Sciences, Division of Surgery, Lund University, Lund, Sweden; Department of Clinical Sciences, Division of Oncology and Pathology, Lund University, Lund, Sweden; Department of Haematology, Oncology and Radiation Physics, Skåne University Hospital, Lund, Sweden; Department of Plastic Surgery, Institute of Clinical Sciences, Sahlgrenska Academy, University of Gothenburg, Gothenburg, Sweden; Department of Plastic Surgery, Sahlgrenska University Hospital, Region Västra Götaland, Gothenborg, Sweden; Department of Clinical Sciences, Division of Surgery, Lund University, Lund, Sweden; Department of Surgery, Skåne University Hospital, Malmö, Sweden; Department of Clinical Sciences, Division of Surgery, Lund University, Lund, Sweden; Department of Plastic and Reconstructive Surgery, Skåne University Hospital, Malmö, Sweden

## Abstract

**Background:**

Postmastectomy radiotherapy (PMRT) impairs the outcome of immediate breast reconstruction in patients with breast cancer, and the sentinel lymph node (SLN) status is crucial in evaluating the need for PMRT. The aim of this study was to develop and validate models to stratify the risk of clinically significant SLN macrometastases (macro-SLNMs) before surgery.

**Methods:**

Women diagnosed with clinically node-negative (cN0) T1–2 breast cancer were identified within the Swedish National Quality Register for Breast Cancer (2014–2017). Prediction models and corresponding nomograms based on patient and tumour characteristics accessible before surgery were developed using adaptive least absolute shrinkage and selection operator logistic regression. The prediction of at least one and more than two macro-SLNMs adheres to the current guidelines on use of PMRT and reflects the exclusion criteria in ongoing trials aiming to de-escalate locoregional radiotherapy in patients with one or two macro-SLNMs. Predictive performance was evaluated using area under the receiver operating characteristic curve (AUC) and calibration plots.

**Results:**

Overall, 18 185 women were grouped into development (13 656) and validation (4529) cohorts. The well calibrated models predicting at least one and more than two macro-SLNMs had AUCs of 0.708 and 0.740, respectively, upon validation. By using the prediction model for at least one macro-SLNM, the risk could be updated from the pretest population prevalence of 13.2% to the post-test range of 1.6–74.6%.

**Conclusion:**

Models based on routine patient and tumour characteristics could be used for prediction of SLN status that would indicate the need for PMRT and assist decision-making on immediate breast reconstruction for patients with cN0 breast cancer.

## Teaser

Postmastectomy radiotherapy (PMRT) impairs the outcome of immediate breast reconstruction in patients with breast cancer, and the sentinel lymph node (SLN) status is crucial in evaluating the need for PMRT. In this study, the authors present two nomograms for evaluation of SLN status before surgery indicating the need for PMRT using clinicopathological characteristics. By offering personalized risk assessments, the models facilitate shared decision-making, guiding discussions on breast reconstruction options, and enabling patients with breast cancer to make more informed decisions about their reconstructive plans.

## Introduction

Breast reconstructive surgery improves the quality of life of patients with breast cancer undergoing mastectomy^1^. According to international guidelines^2,3^, all patients undergoing mastectomy should be counselled about reconstructive options. To evaluate the optimal timing and type of breast reconstruction, risk factors for complications after surgery must be considered, such as smoking^4^, obesity^5^, and diabetes^6^. Specifically, postmastectomy radiotherapy (PMRT) is known to affect outcomes in those receiving immediate breast reconstruction (IBR)^7–10^. Common complications involve capsular contracture and loss of implant following immediate implant-based reconstructions^7,8^; tissue necrosis is also a common complication associated with immediate autologous reconstructions and PMRT^11^. Therefore, evaluating the need for PMRT is essential to facilitate shared decision-making regarding breast reconstructive surgery and IBR in particular.

The decision on PMRT is based on axillary lymph node metastasis, tumour size > 50 mm, or involved resection margins^2,3^. Sentinel lymph node (SLN) biopsy (SLNB) is the standard for axillary nodal staging in patients with clinically node-negative (cN0) invasive breast cancer, and PMRT should be considered in those with at least one SLN macrometastasis (macro-SLNM; > 2 mm), according to current guidelines^2,3^. Ongoing randomized clinical trials (RCTs)^12–14^ are examining the possibility of omitting locoregional radiotherapy in patients with one or two macro-SLNMs. However, for those with a heavy nodal disease burden, PMRT remains advisable. Consequently, the identification of patients with more that two macro-SLNMs is crucial. A non-invasive tool for predicting SLN status before surgery could be beneficial in identifying patients at risk of complications after surgery associated with the requirement for PMRT following IBR. Although there are several nomograms for predicting axillary node status^15–17^, most of these include variables that are not available routinely in a setting preceding surgery. Moreover, predictive tools to identify patients with cN0 breast cancer at high risk of macro-SLNM, indicating the need for PMRT, are still lacking.

This study aimed to develop and validate prediction models for risk stratification of SLN metastasis, indicating the need for PMRT in patients with cN0 breast cancer using only routine clinical patient and tumour characteristics. The primary endpoint (prediction of ≥ 1 macro-SLNM) is directly applicable to current guidelines on the use of PMRT. The secondary endpoint (prediction of > 2 macro-SLNMs) adheres to the exclusion criteria of ongoing RCTs aiming to de-escalate the use of locoregional radiotherapy in patients with a low nodal metastatic burden. To the authors’ knowledge, this is the first study to present prediction models that stratify the risk of macro-SLNMs, indicating the need for PMRT in patients with cN0 breast cancer.

## Methods

### Study population

This retrospective study was conducted using data from the Swedish National Quality Register for Breast Cancer (NKBC), a prospectively maintained, population-based register with almost 100.0% completeness when cross-linked to the Swedish Cancer Register^18^. All women diagnosed with invasive breast cancer in Sweden from January 2014 to December 2017, primarily treated with surgery, were identified. The exclusion criteria were: bilateral breast cancer; neoadjuvant chemotherapy; ductal carcinoma *in situ*; tumour size > 50 mm or unknown tumour size; stage IV breast cancer; palpable axillary lymphadenopathy; absent or incongruent data on axillary nodal status; and omission of SLNB. The data set was split into a development cohort and a validation cohort. The development cohort comprised patients diagnosed in 2014–2016, and the remaining patients constituted the temporal validation cohort.

For all included patients, SLNB was the primary axillary staging procedure for evaluation of axillary nodal status, and nodal metastases were categorized into macrometastases if a metastatic deposit > 2 mm was found^19^. For the prediction of more than two macro-SLNMs, patients with macrometastases in at least two sentinel nodes and with any additional lymph node metastasis identified by SLNB or by completion axillary lymph node dissection were included.

The study was approved by the Swedish Ethical Review Authority (2019–02139) and written informed consent for participation was waived for this register-based study using data from a national quality register that has been collected previously. The study was registered in the ISRCTN database (ISRCTN 14341750) and followed the STROBE guidelines for reporting observational studies^20,21^.

### Clinicopathological predictors

Based on previous studies on variables associated with SLN status^22,23^, the following candidate predictors were evaluated: age at diagnosis; tumour size; histological grade; histological type; oestrogen receptor (ER) status; progesterone receptor (PR) status; amplification of human epidermal growth factor receptor 2 (HER2); and multifocality. Patient age has been reported to have a non-linear association with nodal status, with the lowest risk of lymphatic involvement in those around 70 years of age^24^. Therefore, patients were categorized into three different groups with one group centred around 70 ± 5 years (age groups ≤ 65, 66–75, and > 75 years). Further categorization did not improve the discriminatory ability of the models and was therefore avoided. Multifocality was defined as the presence of at least two foci of invasive breast cancer within the same breast separated by benign tissue on the histological examination of the excised section^25^, and tumour size was defined as the greatest dimension of the largest invasive focus. For ER and PR status, ≥ 1% stained nuclei by immunohistochemistry (IHC) was considered positive according to the definitions of the European Society for Medical Oncology^26^. To evaluate HER2 status, IHC and *in situ* hybridization (ISH) were undertaken, and tumours scoring 3+ on IHC and/or with a positive ISH test were regarded as HER2-positive. The histological type was categorized into three groups: invasive carcinoma of no special type (NST), invasive lobular carcinoma (ILC), and other invasive carcinoma. Mixed types were excluded from the analyses.

### Statistical analysis

Univariable logistic regression analysis was used to explore the unadjusted associations between each candidate predictor and the two endpoints in the development cohort. Adaptive least absolute shrinkage and selection operator (LASSO) logistic regression^27^ was then applied to all candidate predictors to select the most important. LASSO regression is a commonly used machine learning technique for variable selection and model development, minimizing the risk of overfitting by forcing the absolute sizes of the regression coefficients of the standardized predictors to be bounded by a penalty factor, λ. The penalization impairs the importance of each predictor by decreasing the value of the regression coefficients, thus minimizing the risk of overfitting. The adaptive form of LASSO regression leads to even more parsimonious models than the standard LASSO analysis^28,29^. To determine the optimal value of λ, the authors used ten-fold cross-validation in the development cohort. Patients with missing values for one or more of the candidate predictors were removed from the analyses. Nomograms for predicting at least one and more than two macro-SLNMs were developed based on the results of the adaptive LASSO regression analyses.

To evaluate each prediction model’s discriminatory ability, the area under the receiver operating characteristic (ROC) curve (AUC) was calculated in the development and validation cohorts. Model accuracy was assessed in the validation cohort using calibration plots (graphical) and calibration slope and intercept (numerical). To evaluate the clinical utility of the prediction models, decision curve analyses were undertaken on the validation cohort. For comparison purposes, corresponding prediction models based on backward stepwise regression with uniform bootstrap-based shrinkage^30^ were developed (*supplementary methods*). All analyses were done using Stata^®^ release 18 (StataCorp, College Station, TX, USA).

## Results

### Patient and tumour characteristics

**Fig. 1:**
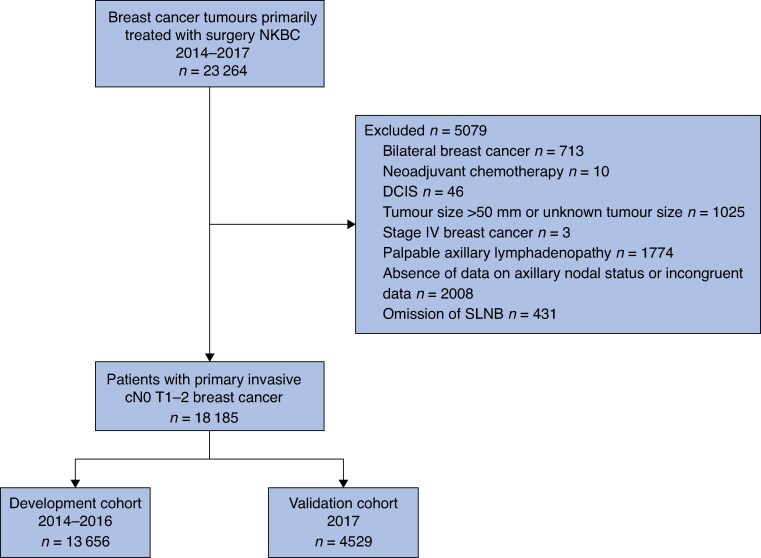
Study flow chart NKBC, Swedish National Quality Registry for Breast Cancer; DCIS, ductal carcinoma *in situ*; SLNB, sentinel lymph node biopsy; cN0, clinically node-negative.

From January 2014 to December 2017, 23 256 breast cancers, primarily treated with surgery, were registered in NKBC (*Fig. 1*). The final study cohort consisted of 18 185 patients with breast cancer who met the eligibility criteria. Of these, 13 656 were diagnosed between 2014 and 2016, constituting the development cohort, and 4529 were diagnosed in 2017, constituting the temporal validation cohort. Patient and tumour characteristics of the development and validation cohorts were comparable (*Table 1*). Overall, 2409 patients (13.2%) had at least one macro-SLNM, and 278 (1.5%) had more than two macro-SLNMs. The overall median age at diagnosis was 64 years, and the median tumour size was 15 mm. Most patients had unifocal, hormone receptor-positive, HER2-negative grade II NST.

**Table 1:** Patient and tumour characteristics of the development and validation cohorts

	All (*n* = 18 185)	Development cohort (*n* = 13 656)	Validation cohort (*n* = 4529)
**Age (years), median (range)**	64 (22–95)	64 (23–95)	65 (22–95)
Missing	0	0	0
**Age (years)**			
≤ 65	9686 (53.3)	7320 (53.6)	2366 (52.2)
66–75	6220 (34.2)	4626 (33.8)	1594 (35.2)
> 75	2279 (12.5)	1710 (12.5)	569 (12.6)
Missing	0	0	0
**Tumour size (mm), median (range)**	15 (1–50)	15 (1–50)	15 (1–50)
Missing	0	0	0
**Histological type**			
NST	13 979 (79.4)	10 511 (79.2)	3468 (79.8)
ILC	2320 (13.2)	1779 (13.4)	541 (12.4)
Others	1315 (7.5)	977 (7.4)	338 (7.8)
Missing	571	389	182
**Histological grade**			
I	4068 (22.6)	3081 (22.8)	987 (22.0)
II	9447 (52.5)	7118 (52.7)	2329 (51.9)
III	4467 (24.8)	3298 (24.4)	1169 (26.1)
Missing	203	159	44
**Multifocality**			
Yes	2924 (16.1)	2167 (15.9)	757 (16.7)
No	15 229 (83.9)	11 463 (84.1)	3766 (83.3)
Missing	32	26	6
**ER status**			
Positive	16 068 (91.8)	12 032 (92.1)	4036 (90.9)
Negative	1432 (8.2)	1028 (7.9)	404 (9.1)
Missing	685	596	89
**PR status**			
Positive	14 637 (84.9)	10 988 (85.6)	3649 (82.9)
Negative	2596 (15.1)	1844 (14.4)	752 (17.1)
Missing	952	824	128
**HER2 status**			
Positive	2009 (11.2)	1497 (11.1)	512 (11.4)
Negative	15 917 (88.8)	11 939 (88.9)	3978 (88.6)
Missing	259	220	39
**Ki-67 (%), median (range)**	20 (0–100)	20 (0–100)	20 (1–100)
Missing	131	98	33
**≥ 1 macro-SLNM**			
Yes	2409 (13.2)	1852 (13.6)	557 (12.3)
No	15 776 (86.8)	11 804 (86.4)	3972 (87.7)
Missing	0	0	0
**> 2 macro-SLNMs**			
Yes	278 (1.5)	203 (1.5)	75 (1.7)
No	17 907 (98.5)	13 453 (98.5)	4454 (98.3)
Missing	0	0	0

NST, carcinoma of no special type; ILC, invasive lobular carcinoma; ER, oestrogen receptor; PR, progesterone receptor; HER2, human epidermal growth factor receptor 2; macro-SLNM, sentinel lymph node macrometastasis.

### Variable selection and prediction model development

The results of the univariable logistic regression analyses are presented in *Table S1*. Only patients with complete information on all candidate predictors were included in the adaptive LASSO logistic regression analyses (12 168, 89.1%). Patient and tumour characteristics for those included in the analyses and those removed because of any missing value for the candidate predictors are presented in *Table S2*. For prediction of at least one macro-SLNM, the adaptive LASSO regression-based prediction model identified patient age, tumour size, multifocality, histological type, histological grade, ER, PR, and HER2 status as predictors. Tumour size emerged as the most important predictor, followed by multifocality (*Fig. S1a*,*b*).

For the prediction of more than two macro-SLNMs, adaptive LASSO regression identified the following predictors: tumour size, histological grade, multifocality, patient age, and histological type (*Fig. S1c*,*d*). For patient age and histological type, only two categories were selected by LASSO regression: > 65 *versus* ≤ 65 years; and ILC *versus* NST or others, respectively. Tumour size was also the strongest predictor in this model.

The penalized regression coefficients from the two adaptive LASSO regression analyses are shown in *Table 2*. Along with the estimated intercept, these coefficients constitute the function used for outcome prediction. A positive coefficient indicates that the variable increases the predicted probability, and a negative coefficient that the variable decreases the predicted probability. The retained predictors and their corresponding regression coefficients in the two supplementary prediction models based on backward stepwise regression with bootstrap uniform shrinkage are presented in *Table S3*.

**Table 2:** Penalized regression coefficients for prediction of at least one and more than two macro-SLNMs identified by adaptive LASSO regression (12 168 patients)

	Penalized coefficients	
	Prediction of ≥ 1 macro-SLNM	Prediction of > 2 macro-SLNMs
**Age (years)**		
≤ 65	0 (reference)	
66–75	−0.276	
> 75	−0.128	
Age (> 65 *versus* ≤ 65 years)		−0.171
Tumour size (mm)	0.068	0.076
**Histological type**		
NST	0 (reference)	
ILC	−0.270	
* *Others	−0.658	
Histological type (ILC *versus* NST or others)		0.070
**Histological grade**		
I	0 (reference)	0 (reference)
II	0.359	0.743
III	0.489	0.813
Multifocality (multifocal *versus* unifocal)	0.643	0.489
ER status (positive *versus* negative)	0.225	
PR status (positive *versus* negative)	0.122	
HER2 status (positive *versus* negative)	−0.078	
Constant	−3.669	−6.451

Binary coding was used for categorical variables with two levels and two binary so-called dummy variables for categorical variables with three levels. Macro-SLNM, sentinel lymph node macrometastasis; LASSO, least absolute shrinkage and selection operator; NST, ductal carcinoma of no special type; ILC, invasive lobular carcinoma; ER, oestrogen receptor; PR, progesterone receptor; HER2, human epidermal growth factor receptor 2.

### Nomogram development

**Fig. 2:**
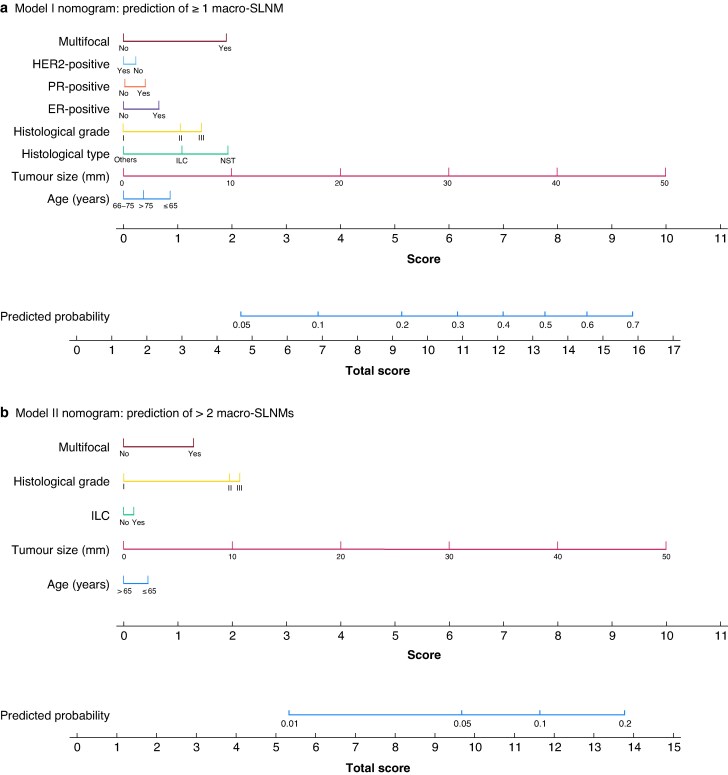
Nomograms predicting SLN status indicating the need for PMRT **a** Probability of at least one sentinel lymph node (SLN) macrometastasis (macro-SLNM) according to current guidelines on use of postmastectomy radiotherapy (PMRT), **b** probability of more than two macro-SLNMs according to endpoints of ongoing clinical trials aiming to de-escalate the use of irradiation. HER2, human epidermal growth factor receptor 2; PR, progesterone receptor; ER, oestrogen receptor; ILC, invasive lobular carcinoma; NST, carcinoma of no special type.

The results from the adaptive LASSO logistic regression analyses were used to develop models I and II with corresponding nomograms, predicting at least one and more than two macro-SLNMs, respectively (*Fig. 2*). The penalized regression coefficients were transformed into specific scores on scales ranging from 0 to 10. By summarizing all scores in one nomogram, the patients’ total score can be applied to a separate scale to predict the presence of at least one and more than two macro-SLNMs.

### Prediction model performance

**Fig. 3:**
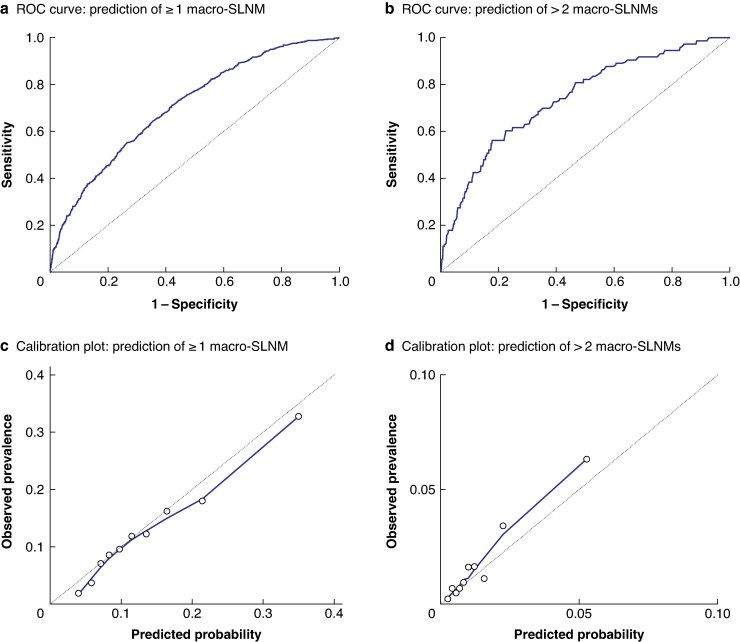
ROC curves and calibration plots Receiver operating characteristic (ROC) curves representing discriminatory ability for **a** model I, predicting at least one sentinel lymph node macrometastasis (macro-SLNM) (area under the curve (AUC) 0.708) and **b** model II, predicting more than two macro-SLNMs (AUC 0.740). Calibration plots illustrating the agreement between the observed prevalence and the predicted probability of **c** at least one macro-SLNM (slope 1.030, intercept –1.08) and **d** more than two macro-SLNMs (slope 0.984, intercept –0.200). These show good calibration, that is the predictions are not systematically biased.

**Fig. 4:**
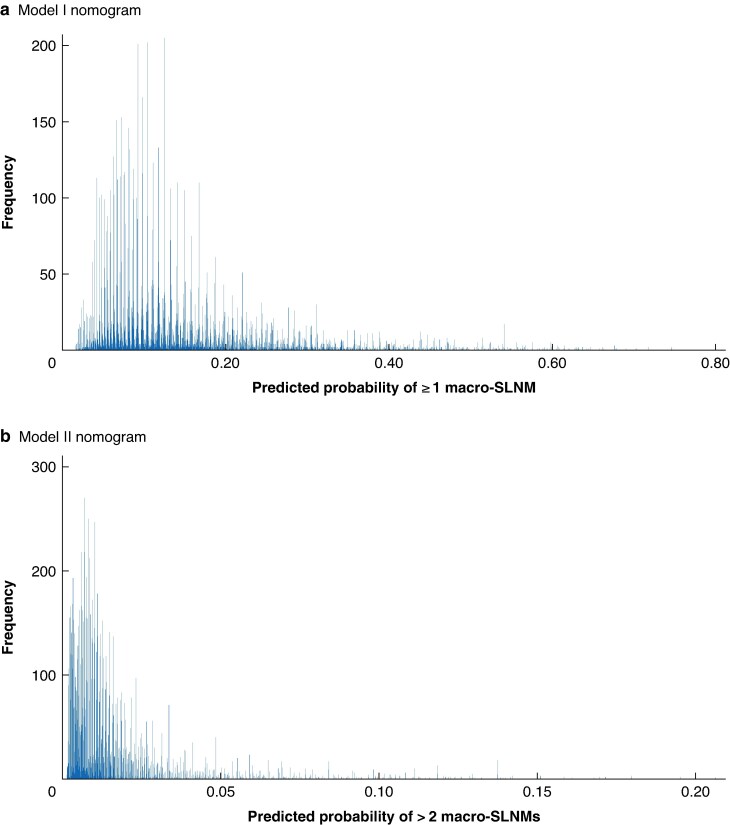
Individual predictions of at least one and more than two macro-SLNMs when applying the nomograms to the overall study cohort **a** Model I: predicted probability of at least one sentinel lymph node macrometastasis (macro-SLNM) ranged from 1.6% for some low-risk patients to 74.6% for some high-risk patients; **b** model II: the predicted probability of more than two macro-SLNMs ranged from 0.1 to 20.6% in the overall study cohort.

The ROC curves and calibration plots illustrating the discriminatory ability and the accuracy of the two prediction models in the validation cohort are presented in *Fig. 3*. For model I, the AUC value was 0.720 (95% confidence interval 0.707 to 0.733) in the development cohort and 0.708 (0.684 to 0.731) in the validation cohort, respectively. As illustrated by the calibration plot, the prediction model displayed good agreement between the predicted and observed prevalence of macro-SLNMs in the validation cohort. The calibration slope and intercept were estimated to be 1.030 and −0.108, respectively. These estimates are close to the optimal values, which are 1.000 and 0 respectively. When applying the nomogram to the overall study cohort, the individually predicted probability of at least one macro-SLNM ranged from 1.6% for some low-risk patients to 74.6% for some high-risk patients (*Fig. 4a*). The results from the decision curve analysis are presented in *Fig. S2a*.

For model II, the AUC values in the development and validation cohorts were 0.775 (0.743 to 0.807) and 0.740 (0.682 to 0.799), respectively (*Fig. 3b*). Similarly, this prediction model was well calibrated with close approximation between the observed prevalence and predicted probability in the validation cohort (*Fig. 3d*), with a calibration slope and intercept of 0.984 and 0.200, respectively. The individually predicted probability of > 2 macro-SLNMs ranged from 0.1 to 20.6% in the overall study cohort, with a mean of 1.4% (*Fig. 4b*). The results from the decision curve analysis are presented in *Fig. S2b*.

AUC values and model calibrations for the two supplementary models based on logistic regression are presented in *Fig. S3*.

## Discussion

This study has developed prediction models and corresponding nomograms based on routine clinical patient and tumour characteristics for risk stratification before surgical treatment of macro-SLNMs, indicating the need for PMRT, which may negatively affect the outcome of IBR. The chosen endpoints are in accordance with the current guidelines on the use of PMRT, and reflect ongoing clinical trials aiming to de-escalate locoregional irradiation in patients with low nodal metastatic burden. The models are well calibrated and can provide individualized estimates of the predicted likelihood of macro-SLNMs before surgery. These models can assist in shared decision-making by helping to assess the clinically relevant risk of macrometastatic nodal involvement, thereby informing discussions about the potential need for PMRT. When applying the nomogram for model I, the predicted risk of at least one macro-SLNM ranged from 1.6% for some low-risk patients to 74.6% for some high-risk patients. By offering personalized risk assessments, the models facilitate shared decision-making, guiding discussions on breast reconstruction options and enabling patients with cN0 breast cancer to make more informed decisions about their reconstructive plans.

To evaluate nodal status indicating the need for PMRT before making a decision on IBR, intraoperative SLN staging was previously undertaken using imprint cytology or frozen sections^31^. However, intraoperative evaluation of SLN status inevitably leaves the patient with some degree of uncertainty before surgery regarding the nodal status and feasibility of IBR. Additionally, it complicates surgical planning, affecting the required time in the operating theatre and the necessary intraoperative resources. Several studies^32–34^ have suggested a staged SLNB procedure before mastectomy when IBR is planned. Although enabling complete pathological evaluation of SLN status before breast surgery, this strategy comes with the drawback of a two-step surgical procedure, including increased risk of infections, delay in definitive surgery, and increased arm morbidity and hospital costs^35,36^. Moreover, the prevalence of lymphatic metastasis at the time of diagnosis is declining^37^, and the number of patients benefitting from an altered treatment plan because of surgically verified macro-SLNM and the need for PMRT is limited^38^. Therefore, use of a complementary, non-invasive prediction tool before surgery would be beneficial when identifying patients for whom IBR is associated with a high risk of complications after surgery because of nodal macrometastasis and PMRT. Ultimately, evaluating the risk factors impairing the outcome of IBR, including the need for PMRT, is essential when supporting shared decision-making regarding breast reconstructive options.

This study has introduced well calibrated models with validated AUCs of 0.708 and 0.740, designed for non-invasive prediction of SLN status, indicating the need for PMRT in patients with breast cancer. Among the previously presented models for the prediction of axillary nodal status in cN0 breast cancer, the Memorial Sloan Kettering Cancer Center nomogram^15^ is one of the most frequently used, with an AUC of 0.754. However, this nomogram includes lymphovascular invasion, which is difficult to obtain before surgery^39^. Similar to most other nomograms for the prediction of axillary nodal status, it does not consider the size of the metastatic deposit. Previous trials^40,41^ have failed to prove any benefit of PMRT for patients with only a micrometastatic deposit (≤ 2 mm), and micrometastatic disease is not included in the current guidelines^2,3^ concerning recommendations for PMRT. Therefore, model I presented here was developed to predict at least one macro-SLNM specifically. The decision curve analysis demonstrates that the prediction model provides a positive net benefit across a wide range of clinically relevant thresholds. The endpoint of the second prediction model was chosen to adhere to the protocols of ongoing RCTs questioning the benefit of locoregional radiotherapy for patients with low nodal metastatic burden and one or two verified macro-SLNMs. In the POSNOC trial^13^, women with T1–2 tumours and one or two macro-SLNMs were randomized to no further axillary treatment *versus* regional radiotherapy/axillary dissection. Similarly, the MA.39^14^ and T-REX^12^ trials investigated the need for regional lymph node radiotherapy in patients with one or two macro-SLNMs; however, these trials were limited to patients with ER-positive, HER2-negative breast cancer. Because radiotherapy is associated with an increased risk of arm lymphoedema^42^, postmastectomy pain syndrome^43^, and cardiopulmonary disease^44^, de-escalation of locoregional radiotherapy would not only improve the outcomes of IBR but also reduce morbidity and non-breast cancer mortality for these patients. Clinical guidelines concerning recommendations for PMRT will need to be revised if the omission of locoregional radiotherapy proves to be non-inferior in these patients, and the importance of identifying metastasis limited to one or two macro-SLNMs will be diminished. To the authors’ knowledge, the model II nomogram is the first to predict more than two macro-SLNMs in patients with breast cancer for whom PMRT will continue to be recommended.

Adaptive LASSO regression was used to develop the prediction models. Although this algorithm has proved to be advantageous in the development of prediction models^45,46^, the adaptive LASSO regression-based models presented in this study did not outperform the prediction models based on backward stepwise regression with bootstrap uniform shrinkage (*supplementary material*). Moreover, the different algorithms showed only small differences in variable selection. This is most likely because of the restricted number of candidate variables recognized as key predictors, so the advantages offered by the adaptive LASSO algorithm were limited.

This study has some limitations. Besides its retrospective nature, all data were obtained from a quality register with incomplete data for some clinicopathological characteristics. However, NKBC is a national, population-based register with high completeness when cross-linked to the Swedish Cancer Register^18^, and good concordance when validated by re-extraction from medical records^47^. Moreover, although many patients were removed from the modelling because of missing data on one or more of the candidate variables, the study still included a large number of observations (18 185). Furthermore, this study is limited by the imbalanced distribution of macro-SLNMs and more than two macro-SLNMs in the overall study cohort. Nevertheless, this is an important observation, highlighting the small number of patients benefitting from altered treatment planning because of pathologically verified macro-SLNMs and the need for PMRT. A strength of this study is the temporal validation of the nomograms regarding both discriminatory ability and model calibration. Even though the models demonstrated better calibration in patients with a lower predicted risk of macro-SLNMs and more than two macro-SLNMs, likely because of the relatively low prevalence of macrometastasis in this cohort, calibration remained acceptable across the risk spectrum. Although these findings suggest that the models could be useful in clinical practice, further validation in larger, independent cohorts is needed to confirm its performance. Although only variables that could be obtained before surgery were included as predictors for nodal disease, the included tumour characteristics were based on the definitive pathology result. Core needle biopsy has been shown to accurately assess the histological type, grade, hormone receptor status, and HER2 receptor status^39,48^. Additionally, although mammography may overestimate or underestimate pathological tumour size^49^ and the presence of multifocality^50^, a previous study^49^ found no significant difference between mammographic measurements before surgery and histopathological sizing after operation. However, it is important to recognize that assessments before surgery may introduce some degree of uncertainty. As a result, the findings should be interpreted with caution, taking into account the potential for variation between estimates before surgery and actual outcomes. To ensure the clinical use of these nomograms, they must be validated in the specific populations they are intended to serve. Moreover, it must be noted that the prediction models do not consider other risk factors for complications after breast reconstructive surgery, such as obesity, smoking, and diabetes.

## Supplementary Material

zraf047_Supplementary_Data

## Data Availability

The data sets generated and analysed in the present study are available from the corresponding author upon reasonable request.
