# Supplementary material for: Development and validation of prediction models for sentinel lymph node status indicating postmastectomy radiotherapy in breast cancer: population-based study
Source: BJS Open. 2025 Apr 8;9(2):zraf047. doi: 10.1093/bjsopen/zraf047 (PMC11977109; doi:10.1093/bjsopen/zraf047)
Supplement: zraf047_Supplementary_Data [file zraf047_supplementary_data.docx]

**Development and Validation of Prediction Models for Sentinel Lymph Node Status Indicating Postmastectomy Radiotherapy in Breast Cancer: Population-Based Study**

Miriam Svensson, MD^1^; Pär-Ola Bendahl, PhD^2^; Sara Alkner, MD, PhD^3^; Emma Hansson, MD, PhD^4,5^; Lisa Rydén, MD, PhD^1,6^; Looket Dihge^.^, MD, PhD ^1,7*^

^1^Department of Clinical Sciences, Division of Surgery, Lund University, Lund, Sweden.

^2^Department of Clinical Sciences, Division of Oncology and Pathology, Lund University, Lund, Sweden.

^3^Department of Hematology, Oncology and Radiation Physics, Skåne University Hospital, Lund, Sweden.

^4^Department of Plastic Surgery, Institute of Clinical Sciences, Sahlgrenska Academy, University of Gothenburg, Gothenburg, Sweden

^5^Region Västra Götaland, Sahlgrenska University Hospital, Department of Plastic Surgery, Gothenborg Sweden.

^6^Department of Surgery, Skåne University Hospital, Malmö, Sweden.

^7^Department of Plastic and Reconstructive Surgery, Skåne University Hospital, Malmö, Sweden.

***** **Corresponding Author:**

Looket Dihge,

Lund University, Faculty of Medicine, Department of Clinical Sciences Lund, Surgery, Medicon Village, SE-223 81, Lund, Sweden

Email: [looket.dihge@med.lu.se](mailto:looket.dihge@med.lu.se), ORCID ID: [**0000-0002-7932-3982**](http://orcid.org/0000-0002-7932-3982), Tel: +46 702556835

**Supplementary Materials - Index**

| **Supplementary Methods** | *page 2* |
| --- | --- |
| **Supplementary Results** | *page 3* |
| **Supplementary Figures and Tables** |  |
| Figure S1 | *page 4* |
| Figure S2 | *page 6* |
| Figure S3 | *page 8* |
| Table S1 | *page 10* |
| Table S2 | *page 11* |
| Table S3 | *page 12* |
| **References** | *page 13* |
|  |  |
|  |  |

**Supplementary Methods**

Univariable logistic regression analysis was used to explore the unadjusted associations between each candidate predictor and the two endpoints in the development cohort. All candidate predictors were entered in a backward stepwise logistic regression analysis using *P* < .157 as the threshold for variable selection. Cases with missing values of ≥1 candidate predictor were removed from the analyses. Two models were developed for predicting ≥1 sentinel lymph node macrometastases (macro-SLNMs) and >2 macro-SLNMs, respectively, based on the results from the logistic regression analysis. Using bootstrapping with 1000 replicates^1^, a uniform shrinkage factor was calculated to shrink each coefficient in the two models, minimizing the risk of overfitting. To evaluate the discriminatory ability for each model, the area under the receiver operating characteristic (ROC) curve (AUC) was calculated. Model calibration was assessed graphically using calibration plots and numerically as calibration slope and intercept.

**Supplementary Results**

The results from the univariable logistic regression analyses are presented in Table S1. Patient and tumor characteristics of those included in the analyses and those removed due to any missing value of the candidate predictors are presented in Table S2. In the multivariable logistic regression analysis, variables that were retained in the final model for ≥1 macro-SLNMs were age, tumor size, histological type and grade, ER status, and multifocality (Table S3). Using bootstrapping with 1000 replicates, the coefficients were modified with a uniform shrinkage factor of 0.988. For the prediction of >2 macro-SLNMs, tumor size, histological grade, and multifocality were retained in the final model after backward stepwise regression. A uniform shrinkage factor of 0.952 was applied to these regression coefficients using bootstrapping.

ROC curves and calibration plots illustrating the predictive performance and accuracy of the two prediction models based on backward stepwise regression with bootstrap uniform shrinkage are presented in Fig. S3. The AUC value for the model predicting ≥1 macro-SLNMs was estimated to be 0.719 (95 per cent confidence interval, 0.707–0.732) in the training cohort and 0.703 (0.680–0.727) in the validation cohort. The model was well-calibrated with a calibration slope and intercept of 1.017 and -0.113, respectively, in the validation cohort. For the model predicting the presence of >2 macro-SLNMs, the AUC in the training and validation cohort was estimated to be 0.773 (0.740–0.805) and 0.742 (0.684–0.800), respectively. Similarly, the model was well-calibrated with calibration slope and calibration intercept of 1.015 and 0.162, respectively.

**Supplementary Figures and Tables**

**Figure S1.** The adaptive LASSO regression algorithm forces the absolute values of the regression coefficients of the standardized predictors to be bounded by a penalty factor λ. Here, the optimal value of λ was determined using 10-fold cross-validation (CV) to minimize the mean deviation across the CV models. Variables with a non-zero coefficient for the optimal value of $\lambda$were selected for the prediction models. (**a**) A λ value of 0.00012, log(λ) = -3.90588 identified (**b**) 11 non-zero coefficients for prediction of ≥1 sentinel lymph node macrometastases (macro-SLNMs), and (**c**) a λ value of 0.00043, log(λ) = - 3.36683 identified (**d**) 6 non-zero coefficients for prediction of >2 macro-SLNMs.

*ILC,* invasive lobular carcinoma; *NST,* no special type; *ER+,* estrogen receptor-positive; *PR+,* progesterone receptor positive; *HER2+,* human epidermal growth factor receptor positive.

**Figure S2.** Decision curve analysis. (**a**) Model I, predicting the probability of ≥1 sentinel lymph node macrometastases (macro-SLNMs), and (**b**) Model II, predicting the probability of >2 macro-SLNMs.

**Figure S3.** Receiver operating characteristic (ROC) curves representing the discriminatory ability for (**a**) prediction of ≥1 sentinel lymph node macrometastases (macro-SLNMs) using backward stepwise regression with bootstrap uniform shrinkage in the temporal validation cohort, and (**b**) prediction of >2 macro-SLNMs. The Calibration plots illustrate the agreement between the observed prevalence and the predicted probability of (**c**) ≥1 macro-SLNMs and (**d**) >2 macro-SLNMs, respectively.

*AUC,* area under the curve.

**Table S1. Univariable logistic regression analyses of clinicopathological characteristics predictive of ≥1 and >2 sentinel lymph node macrometastases (macro-SLNMs) (*n* = 13 656)**

| Variables | Prediction of ≥1 macro-SLNMs | | Prediction of >2 macro-SLNMs | |
| --- | --- | --- | --- | --- |
|  | **OR (95 per cent CI)** | ***P* value** | **OR (95 per cent CI)** | ***P* value** |
| Age (years) |  |  |  |  |
| ≤65 | 1 (reference) |  | 1 (reference) |  |
| 66-75 | 0.709 (0.633 to 0.795) | <.001 | 0.767 (0.553 to 1.064) | .112 |
| >75 | 1.254 (1.090 to 1.444) | .002 | 1.476 (1.017 to 2.142) | .040 |
| Tumor size (mm) | 1.070 (1.064 to 1.075) | <.001 | 1.085 (1.073 to 1.098) | <.001 |
| Histological type |  |  |  |  |
| NST | 1 (reference) |  | 1 (reference) |  |
| ILC | 1.236 (1.077 to 1.418) | .003 | 2.223 (1.600 to 3.087) | <.001 |
| Others | 0.437 (0.337 to 0.568) | <.001 | 0.795 (0.417 to 1.516) | .486 |
| Histological grade |  |  |  |  |
| I | 1 (reference) |  | 1 (reference) |  |
| II | 2.066 (1.779 to 2.398) | <.001 | 3.757 (2.156 to 6.548) | <.001 |
| III | 2.599 (2.212 to 3.053) | <.001 | 4.474 (2.508 to 7.981) | <.001 |
| Multifocality  (multifocal *vs.* unifocal) | 2.043 (1.818 to 2.296) | <.001 | 1.941 (1.417 to 2.658) | <.001 |
| ER status (pos *vs.* neg) | 1.072 (0.887 to 1.295) | .473 | 0.893 (0.541 to 1.475) | .658 |
| PR status (pos *vs.* neg) | 1.084 (0.936 to 1.255) | .280 | 0.859 (0.583 to 1.266) | .442 |
| HER2 status (pos *vs.* neg) | 1.112 (0.954 to 1.295) | .174 | 1.312 (0.876 to 1.963) | .187 |

Binary coding was used for categorical variables with two levels and two binary so-called dummy variables for categorical variables with three levels.

*OR,* odds ratio; *CI,* confidence interval; *NST,* ductal carcinoma of no special type; *ILC,* invasive lobular carcinoma; *ER,* estrogen receptor; *PR,* progesterone receptor; *HER2,* human epidermal growth factor receptor 2.

| Variable | Development cohort  *n* = 13 656 | Included cases  *n* = 12 168 | Removed cases  *n* = 1488 |
| --- | --- | --- | --- |
| Age (years), median (range) | 54 (23-95) | 64 (23-95) | 65 (25-94) |
| Missing | 0 | 0 | 0 |
| Patient age, categories |  |  |  |
| ≤65 years | 7320 (53.6) | 6528 (53.6) | 792 (53.2) |
| 66-75 years | 4626 (33.8) | 4142 (34.0) | 484 (32.5) |
| >75 years | 1710 (12.5) | 1498 (12.3) | 212 (14.2) |
| Missing | 0 | 0 | 0 |
| Tumor size (mm), median (range) | 15 (1-50) | 15 (1-50) | 15 (1-50) |
| Missing | 0 | 0 | 0 |
| Histological type |  |  |  |
| NST | 10 511 (79.2) | 9652 (79.3) | 859 (78.2) |
| ILC | 1779 (13.4) | 1672 (13.7) | 107 (9.7) |
| Others | 977 (7.4) | 844 (6.9) | 133 (12.1) |
| Missing | 389 | 0 | 389 |
| Nottingham histological grade |  |  |  |
| I | 3081 (22.8) | 2840 (23.3) | 241 (18.1) |
| II | 7118 (52.7) | 6466 (53.1) | 652 (49.1) |
| III | 3298 (24.4) | 2862 (23.5) | 436 (32.8) |
| Missing | 159 | 0 | 159 |
| Multifocality |  |  |  |
| Yes | 2167 (15.9) | 1846 (15.1) | 321 (22.0) |
| No | 11 463 (84.1) | 10 322 (84.8) | 1141 (78.0) |
| Missing | 26 | 0 | 26 |
| ER status |  |  |  |
| Positive | 12 032 (92.1) | 11 193 (92.0) | 839 (94.1) |
| Negative | 1028 (7.9) | 975 (8.0) | 53 (5.9) |
| Missing | 596 | 0 | 596 |
| PR status |  |  |  |
| Positive | 10 988 (85.6) | 10 416 (85.6) | 572 (86.1) |
| Negative | 1844 (14.4) | 1752 (14.4) | 92 (13.9) |
| Missing | 824 | 0 | 824 |
| HER2 status |  |  |  |
| Positive | 1497 (11.1) | 1290 (10.6) | 207 (16.3) |
| Negative | 11 939 (88.9) | 10 878 (89.4) | 1061 (83.7) |
| Missing | 220 | 0 | 220 |
| ≥1 macro-SLNMs |  |  |  |
| Yes | 1852 (13.6) | 1647 (13.5) | 205 (13.8) |
| No | 11 804 (86.4) | 10 521 (86.5) | 1283 (86.2) |
| Missing | 0 | 0 | 0 |
| >2 macro-SLNMs |  |  |  |
| Yes | 203 (1.5) | 175 (1.4) | 28 (1.9) |
| No | 13 453 (98.5) | 11 993 (98.6) | 1460 (98.1) |
| Missing | 0 | 0 | 0 |

**Table S2. Patient and tumor characteristics of those included in the multivariable analyses and those removed due to any missing value of the candidate predictors**

Values in parentheses are valid percentages of each column if not otherwise explained. The percentage values are rounded, and the total percentage may, therefore, not be 100.

*NST,* no special type; *ILC,* invasive lobular cancer; *ER,* estrogen receptor; *PR,* progesterone receptor; *HER2,* human epidermal growth factor receptor 2; *macro-SLNMs,* sentinel lymph node macrometastases.

| **Variable** | **Prediction of ≥1 macro-SLNMs** | | **Prediction of >2 macro-SLNMs** | |
| --- | --- | --- | --- | --- |
|  | **Shrinkage coefficients**  **(shrinkage factor = 0.988)** | ***P* value** | **Shrinkage coefficients**  **(shrinkage factor = 0.952)** | ***P* value** |
| **Age** (years) |  |  |  |  |
| ≤65 | 0 (reference) |  |  |  |
| 66-75 | -0.276 (-0.400 to -0.152) | <.001 |  |  |
| >75 | -0.131 (-0.290 to 0.029) | .108 |  |  |
| **Tumor size** (mm) | 0.067 (0.061 to 0.073) | <.001 | 0.072 (0.060 to 0.084) | <.001 |
| **Histological type** |  |  |  |  |
| NST | 0 (reference) |  |  |  |
| ILC | -0.266 (-0.425 to -0.107) | .001 |  |  |
| Others | -0.651 (-0.933 to -0.369) | <.001 |  |  |
| **Histological** **grade** |  |  |  |  |
| I | 0 (reference) |  | 0 (reference) |  |
| II | 0.351 (0.187 to 0.514) | <.001 | 0.837 (0.258 to 1.416) | .005 |
| III | 0.461 (0.276 to 0.656) | <.001 | 0.900 (0.295 to 1.505) | .004 |
| **ER status** (pos *vs* neg) | 0.332 (0.119 to 0.0.546) | .002 |  |  |
| **Multifocality**  (multifocal *vs* unifocal) | 0.635 (0.505 to 0.766) | <.001 | 0.505 (0.172 to 0.838) | .003 |
| **Constant** | -3.643 |  | -6.508 |  |

**Table S3. Regression coefficients of the variables in the two prediction models based on backward stepwise regression with bootstrap uniform shrinkage (*n* = 12 168)**

The constants and coefficients, estimated by the logistic regression algorithm, constitute the function used for predicting the outcome. A positive coefficient indicates that the variable increases the predicted probability and a negative coefficient indicates that the variable decreases the predicted probability. Using bootstrapping, the coefficients have been shrunken to minimize the risk of overfitting. Binary coding was used for categorical variables with two levels and two binary so-called dummy variables for categorical variables with three levels.

*Macro-SLNMs,* sentinel lymph node macrometastases; *NST,* ductal carcinoma of no special type; *ILC,* invasive lobular cancer; *ER,* estrogen receptor.

**REFERENCES**

1. Fernandez-Felix BM, García-Esquinas E, Muriel A, Royuela A, Zamora J. Bootstrap internal validation command for predictive logistic regression models. The Stata Journal. 2021;21(2):498-509.
